# Complex and dynamic transcriptional changes allow the helminth *Fasciola gigantica* to adjust to its intermediate snail and definitive mammalian hosts

**DOI:** 10.1186/s12864-019-6103-5

**Published:** 2019-10-12

**Authors:** Xiao-Xuan Zhang, Krystyna Cwiklinski, Rui-Si Hu, Wen-Bin Zheng, Zhao-An Sheng, Fu-Kai Zhang, Hany M. Elsheikha, John P. Dalton, Xing-Quan Zhu

**Affiliations:** 1 State Key Laboratory of Veterinary Etiological Biology, Key Laboratory of Veterinary Parasitology of Gansu Province, Lanzhou Veterinary Research Institute, Chinese Academy of Agricultural Sciences, Lanzhou, Gansu Province 730046 People’s Republic of China; 2 College of Veterinary Medicine, Qingdao Agricultural University, Qingdao, Shandong Province 266109 People’s Republic of China; 3 National Centre for Biomedical and Engineering Science (NCBES), School of Natural Sciences, National University of Ireland, Galway, Ireland; 4 College of Animal Science and Technology, Guangxi University, Nanning, Guangxi Zhuang Autonomous Region 530005 People’s Republic of China; 5 Faculty of Medicine and Health Sciences, School of Veterinary Medicine and Science, University of Nottingham, Nottingham, UK

**Keywords:** *Fasciola gigantica*, Different developmental stages, Host-pathogen interaction, RNA-sequencing, De novo Transcriptome,

## Abstract

**Background:**

The tropical liver fluke, *Fasciola gigantica* causes fasciolosis, an important disease of humans and livestock. We characterized dynamic transcriptional changes associated with the development of the parasite in its two hosts, the snail intermediate host and the mammalian definitive host.

**Results:**

Differential gene transcription analysis revealed 7445 unigenes transcribed by all *F. gigantica* lifecycle stages, while the majority (*n* = 50,977) exhibited stage-specific expression. Miracidia that hatch from eggs are highly transcriptionally active, expressing a myriad of genes involved in pheromone activity and metallopeptidase activity, consistent with snail host finding and invasion. Clonal expansion of rediae within the snail correlates with increased expression of genes associated with transcription, translation and repair. All intra-snail stages (miracidia, rediae and cercariae) require abundant cathepsin L peptidases for migration and feeding and, as indicated by their annotation, express genes putatively involved in the manipulation of snail innate immune responses. Cercariae emerge from the snail, settle on vegetation and become encysted metacercariae that are infectious to mammals; these remain metabolically active, transcribing genes involved in regulation of metabolism, synthesis of nucleotides, pH and endopeptidase activity to assure their longevity and survival on pasture. Dramatic growth and development following infection of the mammalian host are associated with high gene transcription of cell motility pathways, and transport and catabolism pathways. The intra-mammalian stages temporally regulate key families of genes including the cathepsin L and B proteases and their trans-activating peptidases, the legumains, during intense feeding and migration through the intestine, liver and bile ducts. While 70% of the *F. gigantica* transcripts share homology with genes expressed by the temperate liver fluke *Fasciola hepatica*, gene expression profiles of the most abundantly expressed transcripts within the comparable lifecycle stages implies significant species-specific gene regulation.

**Conclusions:**

Transcriptional profiling of the *F. gigantica* lifecycle identified key metabolic, growth and developmental processes the parasite undergoes as it encounters vastly different environments within two very different hosts. Comparative analysis with *F. hepatica* provides insight into the similarities and differences of these parasites that diverged > 20 million years ago, crucial for the future development of novel control strategies against both species.

## Background

Fasciolosis is an economically important disease of livestock that affects more than 300 million animals (cattle, sheep, water buffalo and goats) [1, 2]. It is recognized by the WHO as an important neglected tropical disease with estimates of between 2.4 to 17 million people infected [3]. The causative agents of this disease are the helminth parasites, *Fasciola gigantica* and *Fasciola hepatica*, commonly referred to as tropical and temperate liver flukes, respectively. *F. gigantica* is widely distributed throughout Asia and Africa [4]. Global economic losses attributed to fasciolosis are estimated to be more than US $3 billion per year [5, 6], while specific losses driven by *F. gigantica* infection are estimated to be in the region of US $ 2.4 billion and US $ 0.84 billion in Asia and Africa, respectively [1].

The *F. gigantica* lifecycle requires two hosts, a snail intermediate host and a mammalian definitive host. Eggs are passed in faeces from the mammalian host into the environment where embryonation is initiated and the parasites develop into miracidia. Following activation by temperature and light, miracidia hatch from the eggs and seek out and infect the snail intermediate host. *F. gigantica* infects a wide range of snail species, including the Eurasian species *Radix auricularia* and *Austropeplea virdis,* and the African species *Radix natalensis* [7]. Within the snail, the parasite progresses through several developmental stages, namely sporocysts, rediae and cercariae, by a process of clonal expansion [8, 9]. Studies by Dinnik and Dinnik [10–12] have shown that *F. gigantica* undergoes five redial generations that result in large numbers of cercariae emerging from the snail. The cercariae settle on vegetation, or remain free flowing in water, and become encysted in a double-layered weather-resistant cyst. These encysted metacercariae infect the mammalian definitive host, typically cattle and buffalo, following their ingestion with contaminated vegetation or water.

The metacercariae double-layered cyst protects the parasite on pasture and ensures its survival following ingestion. Acid proteases from the stomach/rumen remove the outer cyst wall and then following activation at neutral pH by reducing conditions and bile salts within the duodenum the parasites excyst as newly excysted juveniles (NEJ). The NEJ rapidly migrate through the intestinal wall into the liver parenchyma, where they feed extensively on blood and liver tissue to facilitate their dramatic growth and development. The parasites move to the bile ducts, become sexually mature adults and release approximately 20,000 eggs per fluke per day [13] to continue the lifecycle.

A larger proportion of floating metacercariae cysts have been observed for *F. gigantica*, implying that water is an important source of infection in addition to encysted metacercariae on vegetation for *F. gigantica* [5, 14]. Comparative analysis of the maturation and development times of the parasites in the snail host indicates that as a consequence of higher temperatures *F. gigantica* develops more rapidly than its temperate counterpart *F. hepatica* [14]. Furthermore, studies of the infectivity of *F. gigantica* versus *F. hepatica* have shown that certain ruminant species/breeds display different susceptibilities to the two species. For example, Indonesian Thin Tailed sheep have been shown to be highly resistant to *F. gigantica* but susceptible to *F. hepatica* [15]. In contrast, Japanese Saanen goats are more susceptible to *F. gigantica* than to *F. hepatica* [16]. For ruminant species, such as Guirra sheep, that display similar susceptibilities to infection to both species, *F. gigantica* was found to be more pathogenic than *F. hepatica* [17]. The ability of *F. hepatica* to infect a vast range of mammals, including rodents, lagomorphs, ungulates, ruminants, marsupials, camelids and primates [18], suggests universal processes of invasion, virulence and immune modulation that may also play an important role for *F. gigantica*.

In the present study, we have used RNASeq to profile the complex and dynamic transcriptional changes that *F. gigantica* undergoes throughout its lifecycle, when the parasite is exposed to contrasting environmental and host-specific environments. Parsing the data by host, snail intermediate host versus mammalian host revealed that the parasite transcribes distinct subsets of gene families to interact and manipulate each host. Comparative analysis carried out using the available *F. hepatica* datasets highlighted the high level of homology between the tropical and temperate liver flukes while, at the same time, reveals distinct patterns of gene regulation. These new insights into *Fasciola* spp. biology are critical for the future understanding of developmental biology of these major global and economically important parasites and will assist in the development of new vaccine and drug control strategies.

## Results and discussion

### Sequence assembly and functional annotation

Like all digenean trematodes, *F. gigantica* requires two hosts to facilitate its lifecycle, (1) a snail intermediate host where asexual reproduction results in a clonal expansion of the parasite and, (2) a mammalian definitive host where sexual reproduction occurs with the production of large numbers of eggs. Environmental free-living stages link the two hosts; miracidia that emerge from the eggs and immediately seek and penetrate the snail host, and cercariae that rupture from the snail, settle on vegetation or float on water and secrete a coating to form encysted metacercariae that are infective when ingested by the mammalian host.

**Fig. 1:**
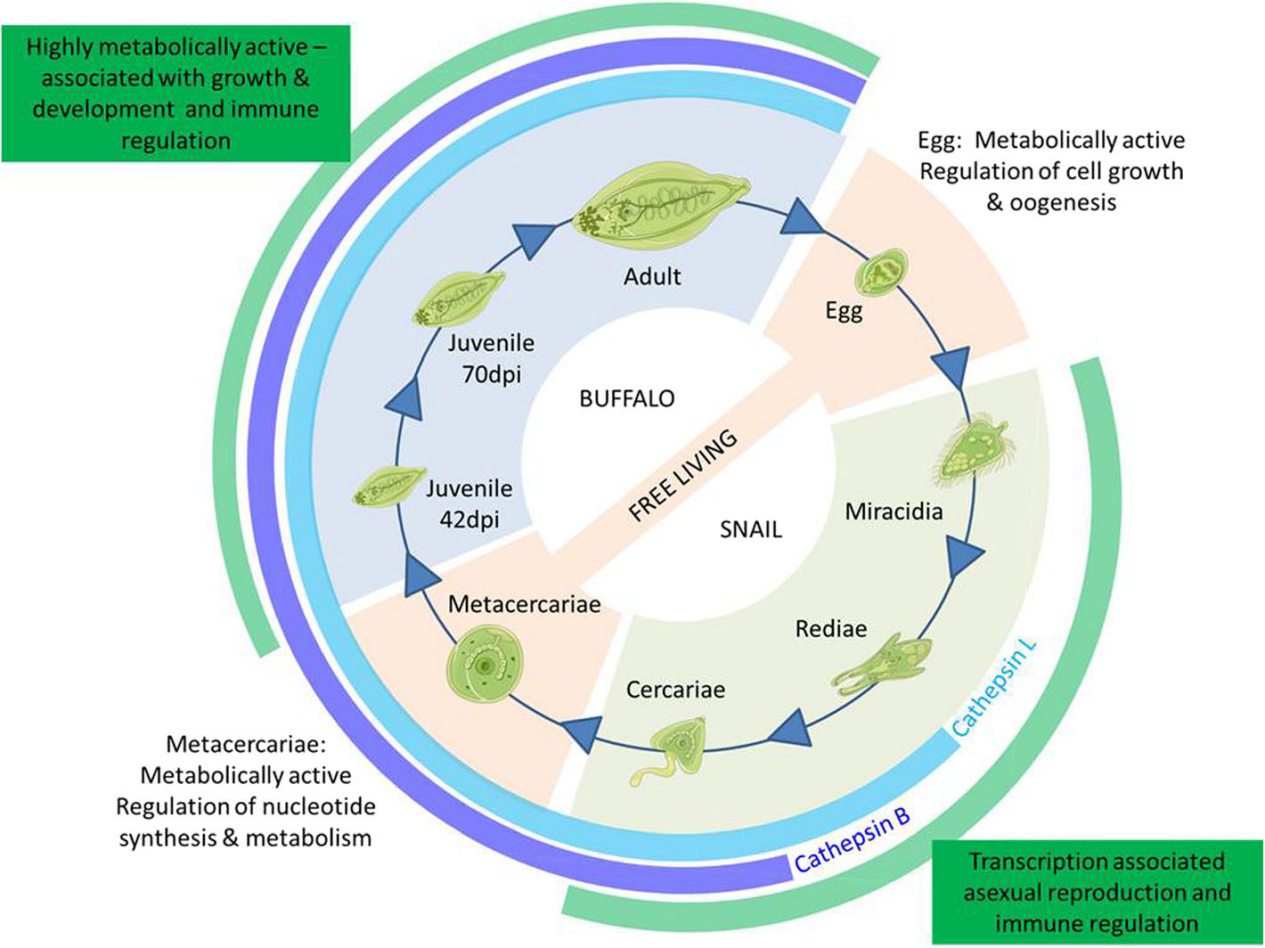
Summary of key metabolic and developmental changes that occur across the lifecycle of *Fasciola gigantica*. Medical art provided by Les Laboratories Servier (https://smart.servier.com/)

To gain insight into how this parasite develops and adapts to these various conditions, we carried out transcriptome analysis of the eight lifecycle stages; eggs, miracidia, rediae, cercariae, metacercariae, liver stage juvenile parasites 42 days post infection (dpi), liver stage juvenile parasites 70 dpi and mature adult fluke taken from the bile ducts (Fig. 1). An average of 54 million high quality reads were generated for each lifecycle stage, which were used for de novo assembly, resulting in 212,790 transcripts (Table 1). The N50 contig size was 1552 nucleotide base pairs, which is consistent with other helminth transcriptomes (*Schistosoma turkestanicum:* 1480 bp [19]; *Eurytrema pancreaticum:* 1094 bp [20]).

**Table 1:** Summary of *Fasciola gigantica* lifecycle stage-specific transcriptome sequence assembly

	Egg	Miracidia	Rediae	Cercariae	Metacercariae	Juvenile-42 dpi	Juvenile-70 dpi	Adult
Raw reads	64061584	47847820	48250358	73797162	49174724	49275028	54126804	56344306
Clean reads	60943084	47847820	46199344	70605896	47239548	46714058	54126804	52842820
GC content	45.62	44.92	44.08	43.37	44.90	45.51	45.04	45.96
Q20 percentage	96.83	95.03	97.27	96.99	96.71	94.96	94.92	94.86
Total unigenes^a^	18197	28697	17854	25902	36293	17518	17187	15635

^a^Clustered unigenes filtered by > 1 FPKM across all three biological replicates for each lifecycle stage

**Fig. 2:**
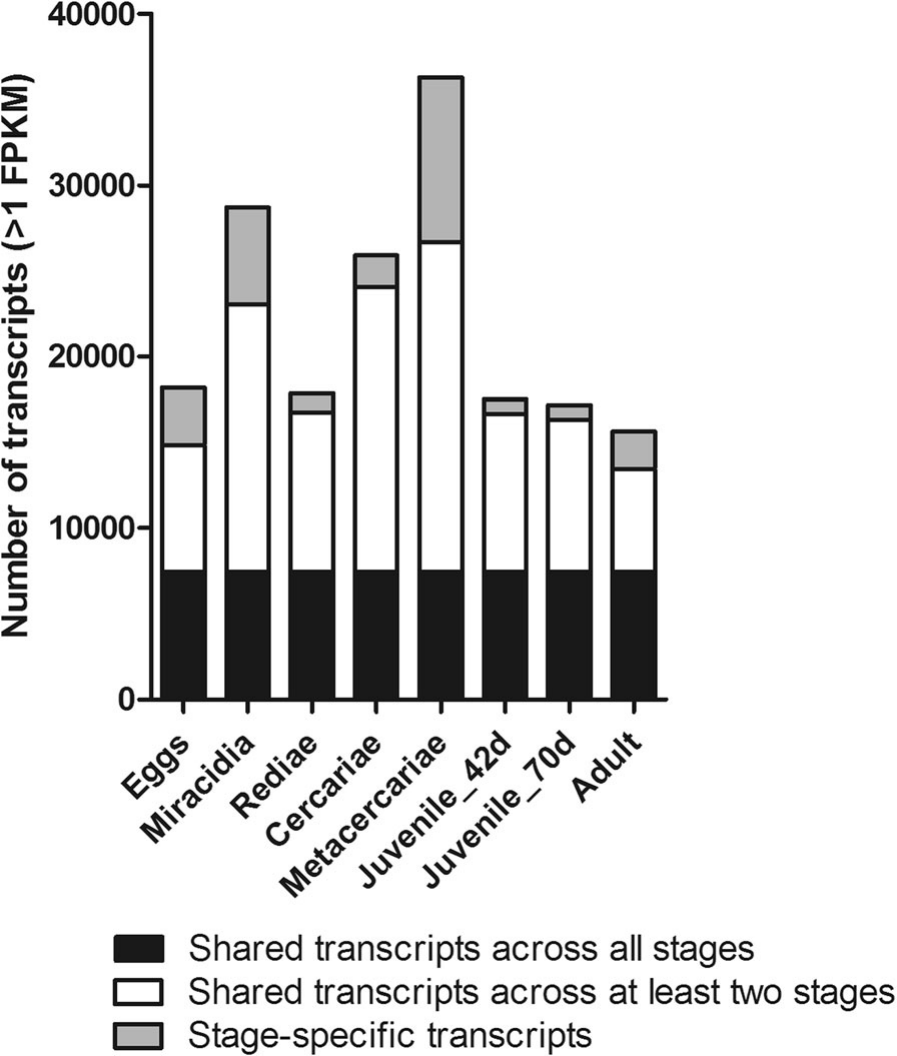
Graphical representation of the *Fasciola gigantica* lifecycle stage-specific transcriptomes. The number of transcripts transcribed by each lifecycle stage is based on the baseline of > 1 FPKM across all three biological replicates. Unigene transcription shared by all eight stages is highlighted in black. Unigene transcription restricted to one lifecycle stage is shown in grey. The remaining transcripts shown in white represent transcription that is shared across at least two stages

The sequencing reads were re-mapped to the stage-specific transcriptome datasets to determine the transcription of the clustered unigenes, and resulted in a total of 58,422 unigenes based on at least 1 fragment per kilobase per million reads (FPKM) in all three replicates across the eight transcriptomes. Analysis of differential transcription revealed that 7445 unigenes were expressed by all eight lifecycle stages (Fig. 2) while the majority (*n* = 50,977) exhibited stage-specific expression. Stage specific expression was observed for each transcriptome dataset (egg, 3372; miracidia, 5646, rediae, 1093; cercariae, 1829; metacercariae, 9605; juvenile-42dpi, 856; juvenile-70dpi, 873; adult, 2192; Fig. 2). Functional annotation of the 58,422 unigene dataset was carried out using seven in silico databases, resulting in 59.2% (*n* = 34,600) of the dataset being annotated by at least one database (Table 2).

**Table 2:** Summary of in silico annotation of *Fasciola gigantica* unigenes (58,422; > 1 FPKM across three biological replicates)

In silico database	Number of transcripts
NCBI Non-redundant (Nr) database	30201 (51.7%)
NCBI Nucleotide (Nt) database	6673 (11.4%)
KEGG (KO) database	11165 (19.1%)
PFAM database	22591 (38.7%)
Gene Ontology	22633 (38.7%)
Biological Process	16554 (28.3%)
Cellular Component	12067 (20.7%)
Molecular Function	18658 (31.9%)
SwissProt database	18783 (32.1%)
KOG database	12316 (22.8%)
Annotated by all databases	1326 (2.3%)
Annotated by at least one database	34600 (59.2%)

### Complexities of the *F. gigantica* lifecycle

#### Eggs and miracidia

**Fig. 3:**
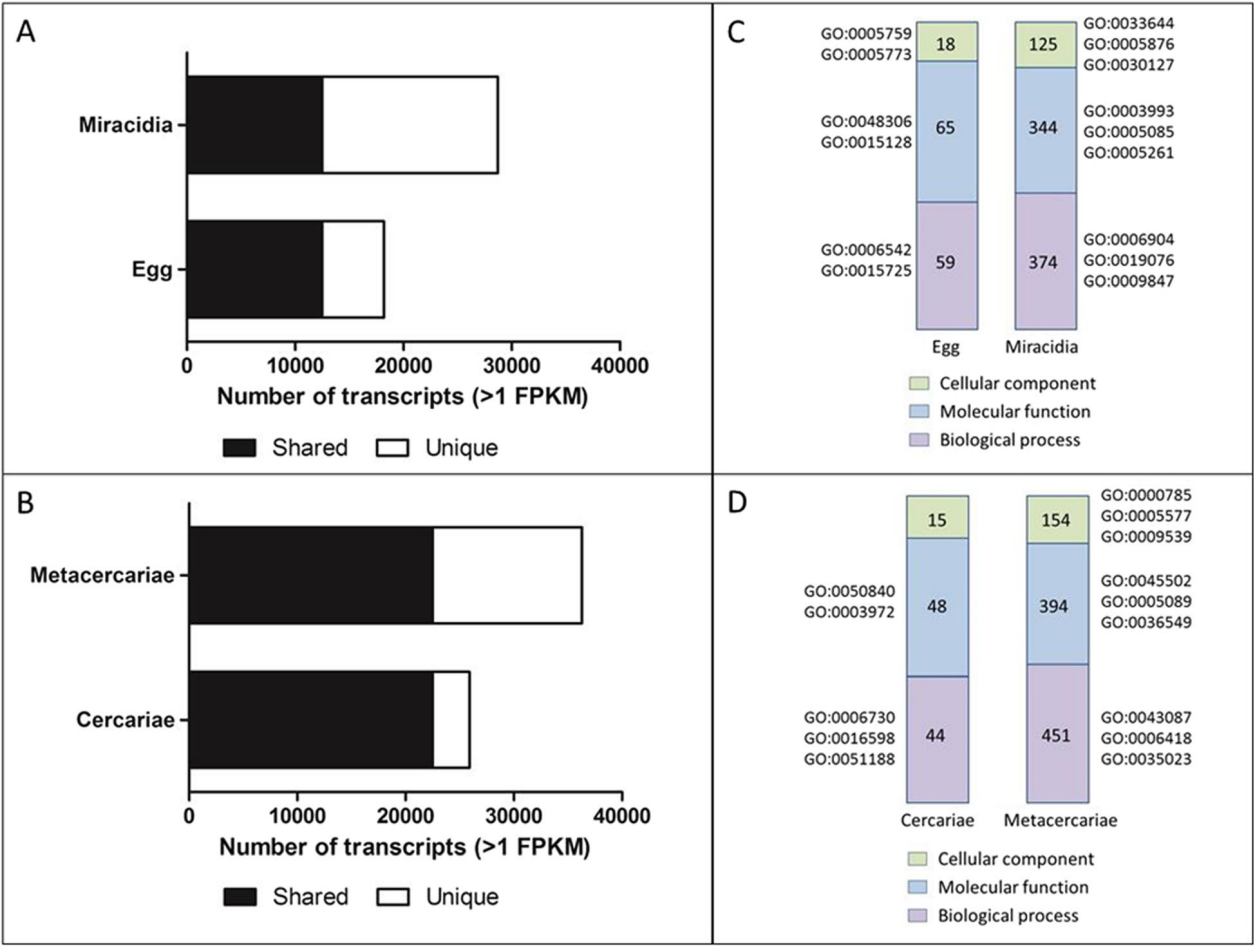
Analysis of the free-living stages, the egg and metacercariae. **a** Graphical representation of the number of transcripts transcribed by the egg and miracidia lifecycle stages (based on at > 1 FPKM across all three biological replicates). **b** Graphical representation of the number of transcripts transcribed by the cercariae and metacercariae lifecycle stages (based on at > 1 FPKM across all three biological replicates). Unigenes transcribed by both stages are highlighted in black and the stage-specific unigenes shown in white. **c-d** Graphical representation of the gene ontology (GO) analysis, highlighting the unique GO terms assigned to each parasite stage (Cellular component GO terms highlighted in green; Molecular function GO terms highlighted in blue; Biological process GO terms highlighted in purple). Key GO terms of interest relating to a high number of transcripts are shown

Eggs passed in the faeces of the mammalian host are undeveloped and require a period of embryonation on pasture under suitable temperature and humidity conditions. Upon embryonation, hatching of the miracidia occurs in response to light and temperature cues [21]. Within the eggs, abundant transcription was observed for transcripts associated with calcium-dependent protein binding (GO:0048306), glycolipid transport and binding activity (GO:0017089, GO:0051861) and gluconate transmembrane transporter activity (GO:0015128). However, transcriptional analysis of the two stages reveals higher levels of transcription in the miracidial stage, with 16,202 transcripts showing stage-specific expression consistent with the high levels of parasite activity (Fig. 3); this difference was also reflected in the higher number of unique gene ontology (GO) terms within the miracidia-specific transcripts (Fig. 3). Key GO terms associated with the miracidia including those related to pheromone activity (GO:0005186) and photosynthesis/light reaction (GO:0019684), and was reflective of the requirement of miracidia to seek out the snail host via light and chemical stimuli [8].

Invasion of the snail host involves penetration and migration through the snail tissues, using both mechanical and proteolytic means [8]. Correlating with the GO enrichment of transcripts relating to catalytic activity upregulated in the miracidia (GO:0003824; Additional file 1: Table S1), a number of transcripts were annotated with the GO term metallopeptidase activity (GO:0008237), which could play a role in the breakdown of snail tissues to facilitate parasite entry. In addition, several GO terms related to extracellular vesicles (EVs) were observed (endocytosis, GO:0006897; vesicle docking involved in exocytosis, GO:0006904) indicating that the miracidia may also use EVs to deliver a cargo of proteases and other molecules for invasion and interaction with the snail host. The GO term alpha-1,6-mannosylglycoprotein 2-beta-N-acetylglucosaminyltransferase activity (GO:0008455) was also found within the miracidia dataset, indicating an essential role in N-glycosylation of proteins in the miracidia which could suggest that parasite glycan-snail lectin interactions are involved in the transformation of miracidium to sporocyst within the snail, as shown in studies of snail interactions with *F. hepatica* [22] and *Schistosoma mansoni* [23].

#### Cercariae and metacercariae

Comparative analysis of the cercariae removed from snails 2 days before shedding with the free-living encysted metacercariae, revealed expression of a greater number of transcripts (n:36,293) associated with the metacercariae and a corresponding greater number of assigned GO terms (*n* = 13,797) demonstrating dramatic changes in gene expression profile (Fig. 2). GO enrichment analysis of the shared transcripts revealed a down-regulation of GO terms associated with nucleotide synthesis and metabolism in the metacercariae consistent with a lower level of metabolic activity (Additional file 1: Table S1). However, since several GO terms were associated with metabolism this indicates that the metacercariae are metabolically active and not completely dormant. Furthermore, 33 biological process GO terms associated with regulation were identified in metacercariae; indicative of their longevity in the external environment this stage expresses genes that regulate (a) gene transcription and translation (transcription from RNA polymerase III promoter, GO:0006359; DNA replication initiation, GO:0032297; DNA transcription, elongation, GO:0032784; translation initiation, GO:0045947), (b) protein phosphorylation (protein kinase activity, GO:0045859), and (c) signal transduction (GO:0009966; ARF protein signal transduction, GO:0032012; Rho protein signal transduction, GO:0035023; GTPase activity, GO:0043087; small GTPase mediated signal transduction, GO:0051056). The encysted parasites appear to maintain their low metabolic state by regulating the pH (GO:0006885) as well reducing their endopeptidase activity (GO:0010951) to avoid autolytic processes.

### Parsing snail and mammalian host transcriptome data

**Fig. 4:**
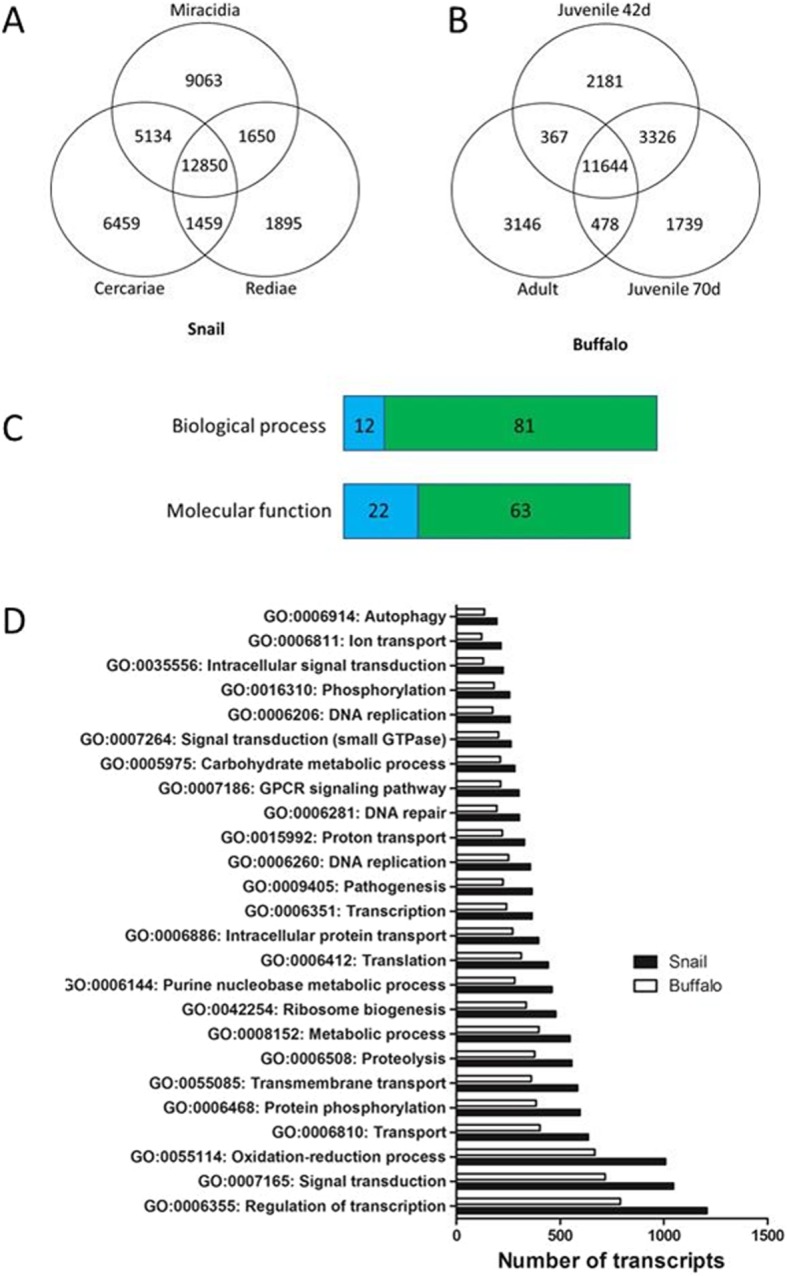
Analysis of host-associated transcription of *Fasciola gigantica* transcripts. **a** Venn diagram representation of the number of transcripts transcribed by the snail intermediate host associated lifecycle stages, namely miracidia, rediae and cercariae. **b** Venn diagram representation of the number of transcripts transcribed by the buffalo mammalian definitive host associated lifecycle stages, namely juvenile 42 dpi, juvenile 70 dpi and adult parasites. **c** Graphical representation of the number of unique gene ontology (GO) terms related to biological process and molecular function transcribed by the snail associated parasite stages, highlighted in green, and the buffalo associated parasite stages, highlighted in blue. **d** Graphical representation of the number of genes annotated with biological process associated GO terms. The terms with the most transcripts assigned are shown. The snail associated parasite stages, shown in black, are compared with the buffalo associated stages, shown in white

The transcriptome data was parsed according to infected host, i.e. stages infecting the snail intermediate host (miracidia, rediae, cercariae) were compared with those in the buffalo definitive mammalian host (juvenile 42 dpi, juvenile 70 dpi, adult) (Fig. 4). A third of the transcripts were transcribed by all the stages within the snail (33.3%; *n* = 12, 850/ 38,510). By contrast, the number of shared transcripts between the buffalo-specific stages represented approximately half of the gene transcripts (50.9%; *n* = 11,644/22,881), reflecting a greater similarity in gene expression between stages in the mammalian stages compared to those within the snail.

Analysis by gene ontology identified the same unique cellular component GO terms in both datasets (*n* = 335). In total 1113 unique biological processes GO terms, and 964 molecular function GO terms were assigned across both datasets. Analysis of those shared by both datasets revealed more genes assigned per GO term within the snail dataset compared with the buffalo dataset (Fig. 4). In addition, 93 biological processes GO terms (snail = 81; buffalo = 12) and 85 molecular function GO terms (snail = 63; buffalo = 22) were associated to only one of the datasets and, therefore, specific to the association with the respective host (Additional file 2: Table S2). The number of unique GO terms particular to the snail associated stages, reflects major biological changes that occur during rapid asexual development in the snail (system development, GO:0048731; tissue development, GO:0009888; regulation of sporulation, GO:0042173; actin cytoskeleton reorganization, GO:0031532; dorsal/ventral pattern formation, GO: 0009953). This contrasted sharply with the buffalo associated stages, where growth is the major developmental change.

Interestingly, several GO terms associated with immune response were identified within the intra-snail stages, including activation of innate immune response (GO:0002218), B cell receptor signaling pathway (GO:0050853), and lymphocyte activation (GO:0046649). This observation is consistent with studies showing that trematode parasites ensure their survival by manipulating the snail’s innate immune system [24–26]. RNASeq analysis of the interaction between *Opisthorchis viverrini* with its snail intermediate host, revealed enrichment of T and B cell signaling pathways and down-regulation of approximately 90% of the differentially expressed genes within infected snails compared with uninfected snail [24].

### Lifecycle stage-specific metabolic regulation

**Fig. 5:**
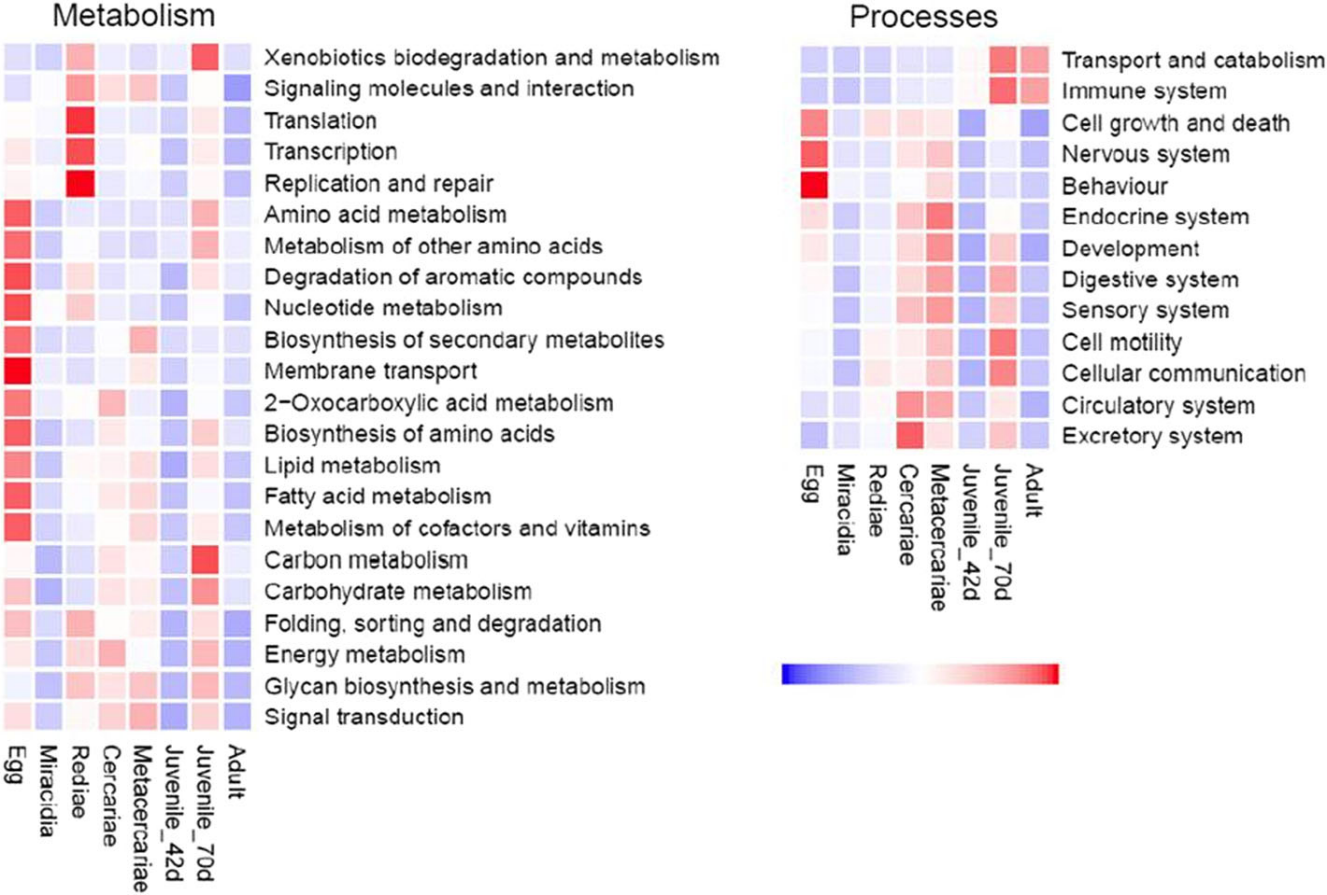
*Fasciola gigantica* metabolism. Graphical representation of the transcription of genes associated with the metabolic KEGG modules (ko00001) relating to metabolism and processes across the *F. gigantica* lifecycle, normalizing the global patterns of expression at the KEGG module level. Relative expression is shown by a blue to red scale depicting low to high levels of transcription, respectively

Analysis of the transcription of the KEGG metabolic pathways reveals that *F. gigantica* displays distinct patterns of metabolic transcription throughout its lifecycle (Fig. 5). Several pathways were highly transcribed by the eggs relative to the other lifecycle stages. In particular, KEGG pathways associated with cell growth and regulation of oogenesis, namely cell cycle (ko04110), oocyte meiosis (ko04114) and p53 signalling pathway (ko04115), which likely play a role in the embryonation process of the eggs on pasture.

Overall, the miracidia show lower relative levels of transcription of the metabolic related genes, particularly relating to pathways involved in carbohydrate metabolism, amino acid metabolism, and transport and catabolism. This is consistent with the short timeframe of this non-feeding stage, as following egg hatching the miracidia must infect a snail within 24 h, with optimal infection success occurring within 2 h of hatching [8]. The swimming rate of the miracidia, which is a determinant for infectivity, is contingent on available glycogen energy stores that are not replenished during this process [8]. Following invasion of the snail the miracidia transform into sporocysts from which the rediae develop. Clonal expansion from the mother redia ensues through daughter redial generations from which the cercariae finally develop [12]. Analysis of redial metabolism revealed that in comparison to the other lifecycle stages, the rediae demonstrated high levels of gene transcription, translation and replication/repair, a requirement for the development of multiple redial generations within the snail. Genes associated with the ribosome (ribosomal proteins); ribosome biogenesis (GTP binding nuclear protein Ran and H/ACA ribonucleoprotein associated protein) and the splicosome (splicing factors and ribonucleoproteins) were amongst the most transcribed genes associated with transcription/translation. In addition, DNA-directed RNA polymerases, transcription initiation factors and translation initiation factors associated with RNA transport also contributed to the high levels of transcription.

The cercariae and metacercariae stages show similar levels of metabolic-related gene transcription. The data reveals that metacercariae regulate the rate of metabolic transcription to remain viable on pasture, which is consistent with similar observations of *F. hepatica* [27]. Overall, the cercariae and metacercariae show comparable levels of metabolic activity to the rediae based on the quantitative metabolic fingerprint values (QMF) of the KEGG metabolism and processes related pathways. However, key metabolic pathways are more abundantly transcribed by the rediae compared with cercariae and metacercariae; for example, gene transcription and translation was between 2.0–3.5 fold increased in the rediae compared with the cercariae and metacercariae. Conversely, a two-fold increase in expression of the transport and catabolism pathways was observed in the cercariae compared with the rediae. Similarly, the biosynthesis of secondary metabolites pathways and membrane transport associated pathways, were more highly expressed by the metacercariae compared to the rediae, at a six-fold and two-fold increased level of transcription, respectively.

Following ingestion by the mammalian host, *F. gigantica* parasites rapidly migrate into the liver parenchyma where feeding begins. In keeping with the rapid growth and development that takes places within the liver tissue, high levels of transcription were observed for the majority of KEGG pathways transcribed by the juvenile 70-dpi parasites. Consistent with the intense feeding and migratory activities of the juvenile parasites through the liver parenchyma, parasites at 42 dpi and 70 dpi transcribed high levels of genes associated with the lysosome and phagosome of the transport and catabolism pathways, and regulation of the actin cytoskeleton related to cell motility pathways. The genes included cathepsin L and cathepsin B proteases, legumains and actin and tubulin genes. Similarly, these genes are also known to be involved in immune system processes, including antigen processing and presentation, platelet activation and leukocyte transendothelial migration, facilitating the parasite’s evasion and manipulation of the host immune response. Glutathione S transferase (mu class), associated with xenobiotics biodegradation and metabolism and glutathione metabolism, was also observed as most abundantly transcribed by the juveniles at 42 dpi and 70 dpi.

In keeping with the increased Krebs cycle activity observed during parasite development that has been shown to be directly correlated to the surface area of the liver fluke parasite [28], pathways involved with carbohydrate metabolism, specifically glycolysis/gluconeogenesis and the citrate cycle (TCA cycle), were highly expressed. Glycan biosynthesis and metabolism pathways, particularly relating to N-glycan biosynthesis, were also observed within the juvenile parasite datasets, consistent with the predominant mannosylated N-glycans found on the outer surface of the *F. hepatica* NEJ and adult parasites [29–31].

Once settled within their final place of residence in the bile ducts, matured adult parasites showed lower relative levels of metabolic transcription compared with the juvenile liver stage parasites. However, the pathways associated with transport and catabolism, namely the phagosome and lysosome (ko04145, ko04142), and pathways associated with the immune system, such as antigen processing and presentation (ko04612), leukocyte trans-endothelial migration (ko04670) and Fc-gamma R mediated phagocytosis (ko04666), are highly transcribed by the adult parasites. Abundant transcription of these KEGG pathways is consistent with the parasites interacting and manipulating the host immune system driving a T helper (Th) 2-type immune response whilst promoting parasite survival through the suppression of pro-inflammatory Th1-type immune responses [32]. Chronic fasciolosis associated with the blood-feeding adult parasites within the bile ducts is characterized by the infiltration of immune cells, including eosinophils, lymphocytes and plasma cells, to the portal areas [33].

### Differential gene expression during the lifecycle of *F. gigantica*

Co-expression cluster analysis of the 58,422 *F. gigantica* unigenes transcribed by the eight lifecycle stages generated 16 clusters that reflect the transitioning gene expression through the lifecycle (Additional file 3: Figure S1). The majority of clusters represented high gene expression at multiple lifecycle stages, with only two clusters (clusters 12 and 15) representing high gene transcription within one lifecycle stage, namely miracidia and adult, respectively. Functional analysis of these clusters revealed that despite different sets of genes being clustered together, key molecular processes are enriched throughout the lifecycle and are present within multiple clusters. In particular, several GO terms were significantly enriched throughout the eight lifecycle stages associated with processes involving nucleic acids (GO:0000166;GO:0003723; GO:0003724; GO:0003676; GO:0003677; GO:0006310), transcription and translation (GO:0003743; GO:0006355; GO:0006446) and energy (ATP) regulation (GO:0005524; GO:0016887; Additional file 4: Table S3). Key GO terms associated with liver fluke biology, including catalytic activity (GO:0003824) and proteolysis (GO:0006508), were also significantly enriched in all eight lifecycle stages. Consistent with a recent study by McVeigh and colleagues of G protein-coupled receptors within the *F. hepatica* genome [34], the GO term G-protein coupled receptor signaling pathway (GO:0007186) was also highly enriched throughout the lifecycle.

In addition to the enriched processes observed throughout the lifecycle, several GO terms were uniquely enriched to particular clusters. Cluster 1 that represents high gene expression from the egg stage through to the metacercariae showed enrichment for metalloendopeptidase activity (GO:0004222) and vesicle-mediated transport (GO:0016192), consistent with our in depth analysis of the snail associated lifecycle stages. Cysteine-type endopeptidase activity (GO:0004197) and cysteine-type peptidase activity (GO:0008234) were significantly enriched within the highly expressed genes within the stages associated with the mammalian host (metacercariae through to adult fluke; cluster 5). By contrast, the only GO term significantly enriched within clusters 11 and 12 that show high levels of expression within the miracidia and cercariae is zinc ion binding (GO:0008270). Studies of the effects of heavy metals and pesticides by Falis and colleagues [35] have shown that zinc may have a protective effect on the survival of miracidia, which may reflect the high levels of genes associated with zinc ion binding. Similarly, the co-enrichment of zinc ion binding and metallo-endopeptidase activity within the eggs and miracidia is consistent with the presence of these zinc-dependent peptidases within these stages.

**Fig. 6:**
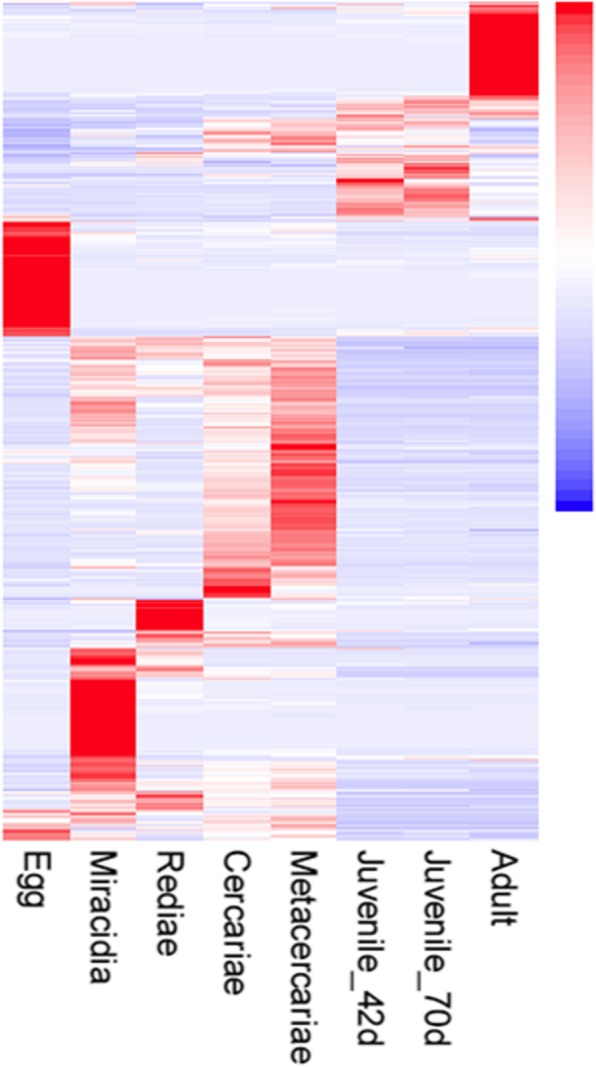
Differential gene expression of the most abundant transcripts across the *Fasciola gigantica* lifecycle. Transcripts representing three biological replicates per lifecycle stage that shown at least a 16 fold difference in transcription between at least two lifecycle stages (9468 unigenes with > 1 FPKM) were grouped by hierarchical clustering, represented by a heatmap (upregulation represented in red; downregulation represented in blue). Abundant stage-specific transcription is highlighted by the red clusters

Deeper analysis across the lifecycle transcriptomes revealed that a total of 34,264 transcripts were significantly differentially expressed (based on at least 1 FPKM across three replicates; Additional file 5: Table S4). In particular, 9468 DEG were found to display at least a 16-fold difference in transcription between at least two of the lifecycle stages. Hierarchical clustering of these most abundant transcripts demonstrated stage-specific expression (Fig. 6), consistent with the developmental regulation of genes associated with metabolism. Analysis of 661 molecular function associated GO terms assigned to the 9468 DEG revealed high representation of GO terms associated with protein binding (GO:00005515), DNA binding (GO:0003677), ATP binding (GO:0005524) and ion binding (GO0008270: zinc ion binding; GO:0046872: metal ion binding; GO:0005509: calcium ion binding) (Additional file 9: Table S7). In addition, key GO terms were highly represented within stage-specific clusters, e.g. GTPase activity (GO:0003924) and microtubule activity/binding (GO:0003777, GO:0008017) were highly represented within the adult parasites. Consistent with the known reliance of *Fasciola* spp. on cathepsin cysteine proteases for migration and feeding in the mammalian host, transcription for these were particularly abundant in the metacercariae and liver stages at 42dpi and 70dpi.

**Fig. 7:**
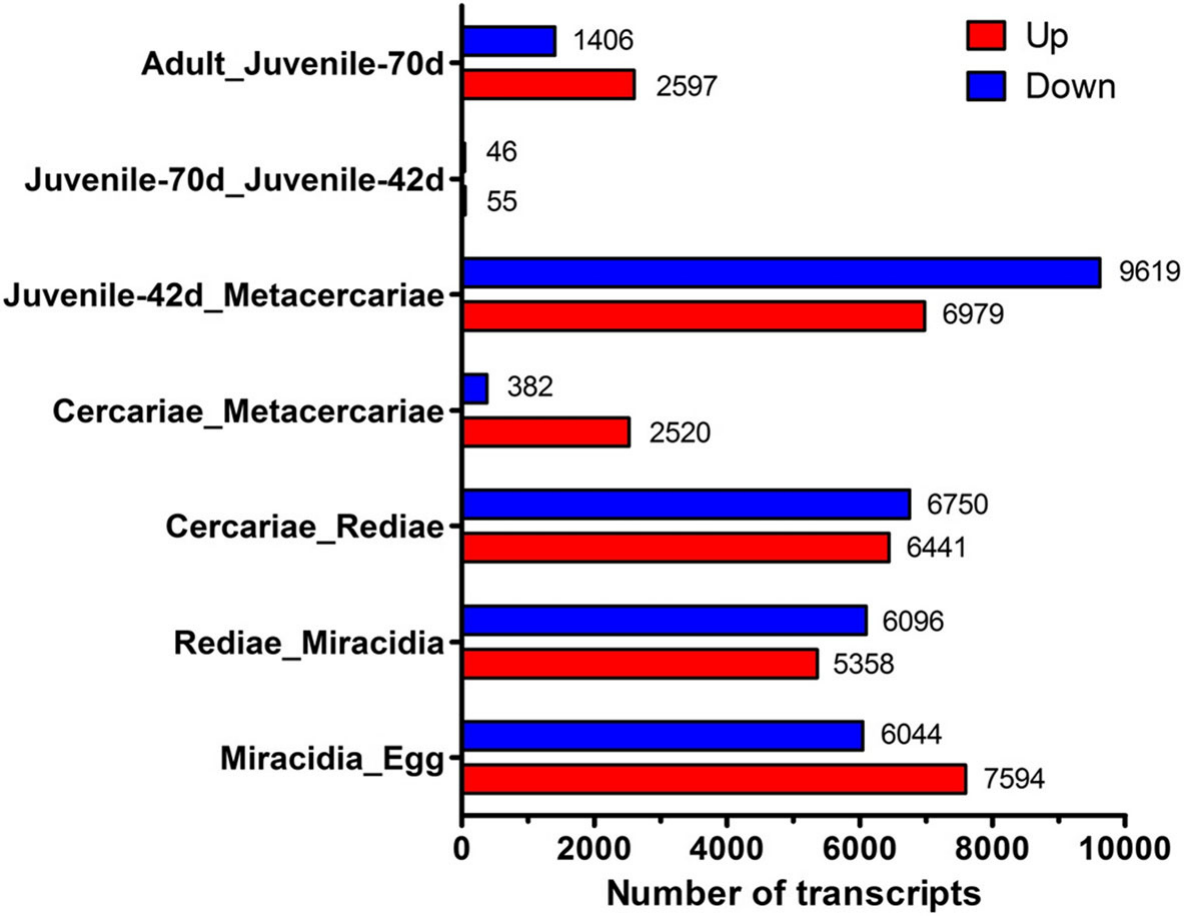
Pairwise analysis of the eight *Fasciola gigantica* transcriptomes. Graphical representation of the number of statistically significant differentially expressed transcripts

To further investigate how the parasites changes and develops through each lifecycle stage, we carried out pairwise DEG analysis of the sequential lifecycle stages, as follows (Fig. 7); (1) egg versus miracidia, (2) miracida versus rediae, (3) rediae versus cercariae, (4) cercariae versus metacercariae, (5) metacercariae versus juvenile 42 dpi, (6) juvenile 42 dpi versus juvenile 70 dpi and (7) juvenile 70 dpi versus adults. Gene ontology enrichment analysis revealed key processes were enriched within each pairwise analysis (Additional file 1: Table S1). As an example, cathepsin L and B, legumain, GST (sigma class) and protease inhibitors (cystatins, serpins and kunitz-type serine protease inhibitors) were observed to be amongst the most abundant unigenes transcribed by the rediae, cercariae, metacercariae, juvenile parasites and adults.

Transcripts upregulated in the rediae and cercariae were enriched for GO terms relating to binding, particularly protein binding, ATP binding, DNA binding, metal ion including zinc ion binding, indicative of a parasite that is metabolically highly active. Heat shock proteins, a range of protease inhibitors (serpins, cystatins and kunitz-type inhibitors) and egg related proteins (yolk ferritin and vitelline protein) were amongst the most upregulated unigenes transcribed by the eggs. Cubilin-related genes were the most represented genes within the miracidia upregulated unigenes.

Compared to the infective metacercarial stage, the juvenile 42dpi parasites displayed abundant transcription of genes relating to metabolic process (GO:0008152) and catalytic process (GO:0003824), reflecting the active feeding and growth of the juvenile parasites when they enter the liver. However, migrating juveniles at 42 dpi and 70 dpi shared a similar transcriptional profile with no overall enrichment indicating little difference in the transcriptional activity of parasites that are slowly moving through the parenchyma mass through their tunneling activity and evading the infiltration of immune cells into damaged sites. Single genes annotated as cathepsin L, legumain, thioredoxin and glutathione S-transferase (GST) sigma class were identified, and are clearly relevant to the immunopathology associated with acute fasciolosis.

Consistent with recent analysis of the early migrating stages of *F. hepatica* [27], we observed expression and temporal regulation of a family of transmembrane water channels, the aquaporins, which facilitate the transport of water and other solutes. Gene transcripts were observed for both the aquaporins, that enable the passage of water, in addition to a subset of channels within this family that also allow the transportation of other solutes including urea and glycerol, termed the aquaglyceroporins based on the signature Froger’s residues and the residues comprising the aquaporin aromatic/arginine selectivity filter [36–38]. These conserved residues were also comparable with those observed within the *F. hepatica* aquaporin sequences [27]. Differential transcription of these genes was observed across the lifecycle, with one subset of aquaporins being expressed almost exclusively by the free-living eggs and miracidia while other aquaporin genes were more highly expressed during the stages present within the mammalian host. Equally, the aquaglyceroporins genes were predominately expressed by the migrating juvenile (42dpi and 70dpi) and the adult parasites, with some genes also being transcribed by the rediae stage. This regulation of surface transport molecules highlights a molecular system by which the parasites adjust to the varying osmotic conditions found between the external environments and the two different hosts the parasite infects.

Regulation of the complement membrane attack complex (MAC) CD59-like genes across the snail- and mammalian-associated stages was also observed, implying that the parasite uses host mimicry to evade and manipulate the immune systems of its hosts. Nine CD59-like genes have been identified within the *F. hepatica* genome [39] indicating that both *Fasciola* species may use similar methods to inhibit host complement.

### Comparative analyses between *F. gigantica* and *F. hepatica*

Levels of homology between the available sequences for *F. gigantica* and *F. hepatica* were determined by reciprocal nucleotide BLAST analysis (*F. gigantica*, *n* = 58,422, present study; *F. hepatica*, *n* = 22,676, derived from the *F. hepatica* genome [40]). Homologous sequences were observed for approximately 71% of the *F. gigantica* transcripts (*n* = 41,355), which included 34,868 transcripts (~ 60% transcripts) that shared nucleotide homology at BLAST e value < e^− 30^. Our results are consistent with the analysis by Young and colleagues who compared the transcriptomes of adult *F. gigantica* and *F. hepatica* [41]. However, it is important to note that the gene models from the *F. hepatica* genome were determined based on transcriptome data from stages from the mammalian host and did not include potential snail-associated lifecycle stage-specific genes [40].

Further analysis was carried out between the lifecycle stages for which transcriptome data was available for both *Fasciola* species, namely egg, metacercariae and adult flukes. Gene ontology analysis of the respective transcriptomes revealed that approximately 900 GO terms were shared by the two *Fasciola* species (egg: 862; metacercariae: 931; adult: 903; Additional file 6: Table S5). However, the majority of significantly enriched GO terms showed species-specific GO enrichment namely within the *F. gigantica* datasets (Additional file 6: Table S5). Key GO terms identified include cysteine-type endopeptidase inhibitor activity (GO:0004869), threonine-type endopeptidase activity (GO:0004298) and positive regulation of catalytic activity (GO:0043085) that were enriched within the *F. gigantica* egg transcriptome and mannose metabolic process (GO:0006013), glycolytic process (GO:0006096) and ferric iron binding (GO:0008199) for the *F. gigantica* adult transcriptome.

Similarly, an abundance of GO terms were identified that were unique to only one of *Fasciola* species, with the majority of significantly enriched GO terms only being identified within the *F. gigantica* egg and adult datasets (Additional file 6: Table S5). Consistent with *F. gigantica* using blood as a food source to facilitate the production of thousands of eggs daily [13], significant GO term enrichment was observed within the *F. gigantica* egg and adult transcriptomes, particularly pertaining to iron and heme transport and processes (GO:0015684: iron ion transport; GO:0015093: ferrous iron transmembrane transporter activity; GO:0006783: heme biosynthetic process). In addition, consistent with the adult parasites being sexually mature, significant enrichment was observed for GO terms relating to processes involving hormones (GO:0008207: C21-steroid hormone metabolic process; GO:0008209: androgen metabolic process; GO:0008210 estrogen metabolic process).

Double clustering analysis was carried out on the genes transcribed by *F. gigantica* and *F. hepatica* eggs, metacercariae and adults to investigate whether these three lifecycle stages show comparable levels of gene transcription (Additional file 7: Figure S2). Twelve clusters were generated, representing a total 36,990 genes (*F. hepatica*: 8511; *F. gigantica*: 28,479) based on gene transcription by the three lifecycle stages. GO enrichment analysis revealed that across all the clusters the majority of the significantly enriched terms were associated with gene transcription and translation (Additional file 8: Table S6). Reflecting the distinctly different stages of the parasite, the clustering analysis revealed that the parasites transcribe different genes at the different time-points of the lifecycle. Specifically, clusters 6 and 7 represents genes transcribed by the adult parasites, with enrichment for unique GO terms associated with peptidase inhibitor activity (GO:0030414), aspartic-type endopeptidase activity (GO:0004190) and G protein-coupled receptor signaling pathway (GO:0007186). High levels of gene transcription within the metacercariae are represented by clusters 9–11, which display enrichment for unique GO terms associated with small GTPase mediated signal transduction (GO:0007264), regulation of transcription (GO:0006355) and translation initiation factor activity (GO:0003743).

Only three clusters contained comparable numbers of *F. hepatica* and *F. gigantica* genes (cluster 4, cluster 5, cluster 9), indicating that despite high levels of homology at the gene sequence level (~ 70%; as above), this is not reflected in the transcription of these genes. Our analysis revealed low numbers of homologous genes within each of the clusters, ranging from 38 transcripts (cluster 10) to 228 transcripts (cluster 8) (Additional file 7: Figure S2), further highlighting the differences in levels of gene transcription between the two *Fasciola* species.

**Fig. 8:**
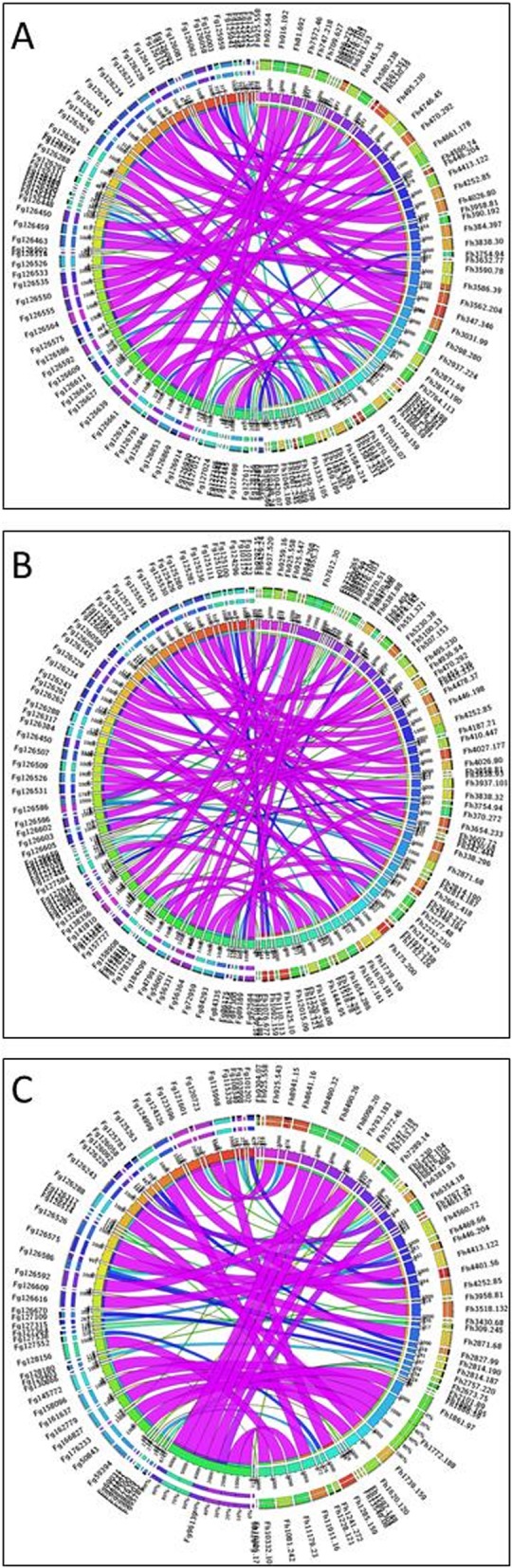
Comparative analysis between *Fasciola hepatica* and *Fasciola gigantica*. Circos diagrams illustrating the best reciprocal BLAST hits of the most abundantly expressed transcripts between *F. hepatica* and *F. gigantica* with the transcriptomes of (**a**) egg, (**b**) metacercariae and (**c**) adult. The width and colour of the ribbons genes is determined by the BLAST e-value score, (Additional file 10: Table S8); narrow to wide ribbon, and a colour change through red-green-blue-purple, indicating a low to high scoring match. Full transcripts names are included in Additional file 10: Table S8

To investigate whether the differences in gene transcription was reflected in the most abundantly expressed genes, in depth reciprocal BLAST analysis was carried out on 250 transcripts from each *F. gigantica* lifecycle stages to identify the homologous sequences within *F. hepatica* (Additional file 10: Table S8). Although these genes represent between 1 and 2% of the total number of genes transcribed by each of the *F. gigantica* lifecycle stages, based on the level of the transcription (FPKM) they represent 74.4, 49.8 and 81.4% of the total transcription of the egg, metacercariae and adult transcriptomes, respectively. With the exception of 22 genes, homologous sequences were identified for all the *F. gigantica* transcripts across the three life-cycle stages. Moreover, these sequences show a high level of nucleotide sequence identity with their *F. hepatica*-specific counterpart (> 80% nucleotide sequence identity; e value <e^− 20^; Fig. 8). Consistent with the cluster analysis (Additional file 7: Figure S2), these homologous sequences did not show comparable levels of expression, with only 75 sequences showing similar levels of expression within both the *F. hepatica* and *F. gigantica* datasets. This comparative data suggests species-specific differential gene regulation that warrants further investigation to determine if this is related to intermediate/definitive host specificity.

Analysis of the 250 genes abundantly expressed by both species highlighted key molecules of biological interest. Eggs were found to abundantly express yolk ferritin, aspartic protease and heat shock proteins, as well as redox based anti-oxidants such as peroxiredoxin (Prx) and glutathione S transferase (GST)-sigma class typically found in dormant/encysted parasite metacercarial stages [27, 42–45]. Consistent with studies of the mammalian infective stage [27, 46], transcripts annotated as cathepsin L and B, and legumain, in addition to redox based anti-oxidants Prx, fatty acid binding protein (FABP) and superoxide dismutase (SOD) were highly expressed within the metacercariae. While similar genes were identified with the adult-specific transcripts, this stage also highly expressed cysteine peptidase inhibitors (cystatin) and the potent immunomodulatory protein termed helminth defense molecule (FhHDM [47]).

### Regulated transcription of the *F. gigantica* cathepsin L and B gene families

Consistent with our analysis of the *F. hepatica* genome and developmental stage-specific transcriptomes, we identified many *F. gigantica* transcripts annotated as cathepsin L and B peptidases, in addition to their *trans*-activators, the asparaginyl endopeptidases (legumains) [48]. Unlike other helminths, *Fasciola* spp. rely almost exclusively on cathepsin L and cathepsin B like cysteine proteases to facilitate tissue migration, feeding, and immune evasion/modulation [49, 50]. Analysis of the recently sequenced *F. hepatica* genome has confirmed that the large cathepsin L gene family has arisen following gene duplication, which based on current genome assemblies is comprised of 23 genes, followed by functional divergence [51]. In addition, a family of 11 cathepsin B gene sequences was identified within the *F. hepatica* genome that arose in similar fashion [51]. Our knowledge of *F. gigantica* cathepsin gene family structure has largely been inferred from studies of *F. hepatica*. However, 16 *F. gigantica* cathepsin L sequences, which have been predominantly identified as cathepsin L1, and seven *F. gigantica* cathespin B sequences are publically available via GenBank. In addition, further sequences, identified as part of an RNAseq project of adult *F. gigantica* parasites isolated from water buffalo at an abattoir in Thailand, include three cathepsin L-like sequences and 17 cathepsin B-like sequences [41].

**Fig. 9:**
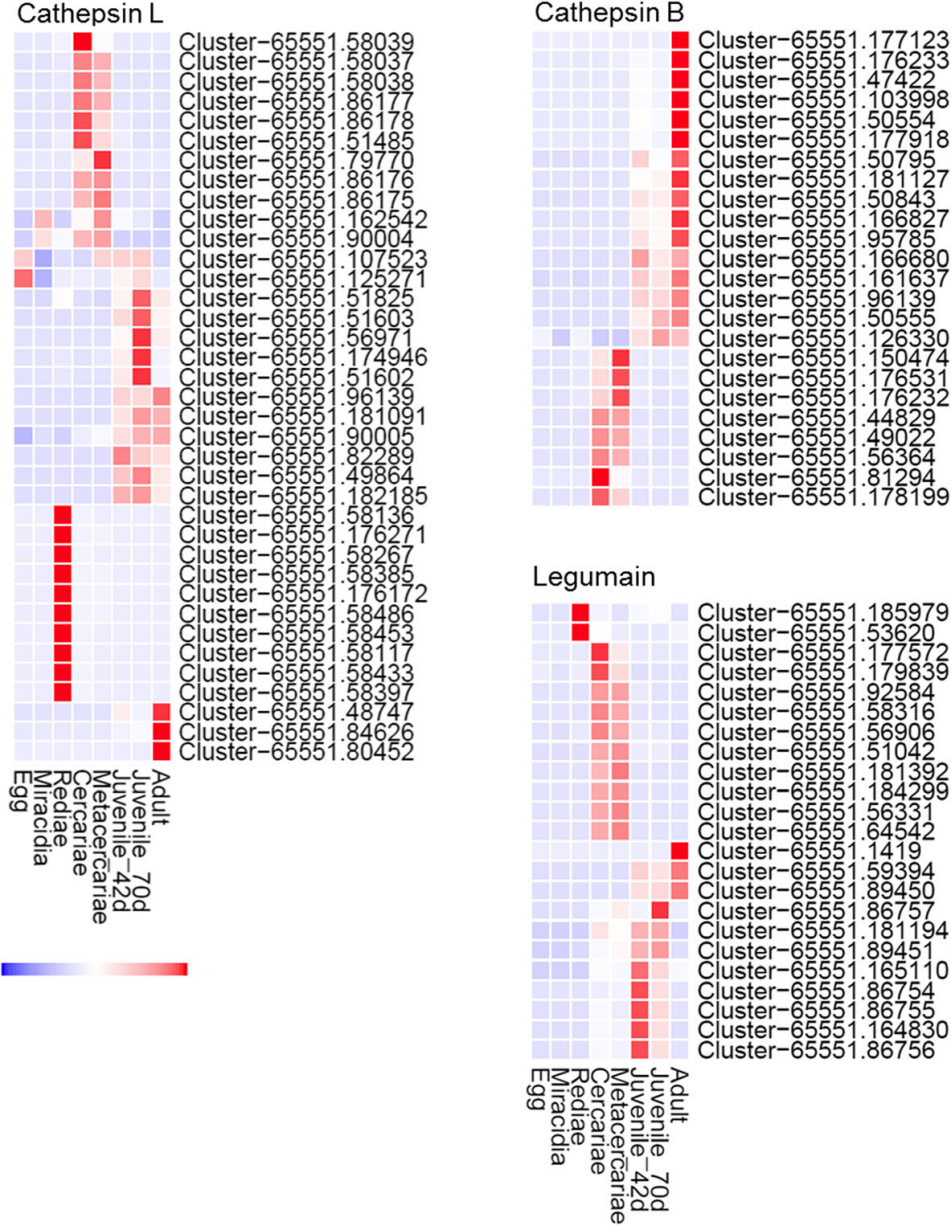
Transcriptional profile of cathepsin cysteine proteases and legumains over the lifecycle of *Fasciola gigantica*. Graphical representation of the transcription of cathepsin L, cathepsin B and legumain transcripts, grouped by hierarchical clustering, represented by a heatmap (upregulation represented in red; downregulation represented in blue)

Interrogation of the eight *F. gigantica* transcriptomes presented in this study using the available *F. hepatica* cathepsin and legumain sequences identified 37 transcript clusters as cathepsin L sequences, 24 transcript clusters as cathepsin B sequences and 23 transcript clusters as legumain sequences (Fig. 9). Hierarchical clustering analysis has shown that similar to *F. hepatica*, the *F. gigantica* cathepsins are also strictly developmentally regulated (Fig. 9). In addition to a group of cathepsin L genes abundantly expressed by the liver migratory stages and the adult parasites (likely homologous to *F. hepatica* cathepsins L1, L2, L3 and L5), several cathepsin L sequences were abundantly expressed by the snail rediae stage. By contrast, we observed that eggs, miracidia and rediae do not abundantly express cathepsin B-like peptidases, indicating that this peptidase class plays a less central role in migration in the snail despite being critical for the mammalian host life-cycle stages.

Of further interest was the observation that the legumain genes are abundantly transcribed, from the intra-snail redial stages through to the adult life-cycle stages (Fig. 9). This is consistent with the abundant expression of the various cathepsin genes, but also implies that these trans-activating peptidases are fundamental in regulating the proteolytic activity of the parasite throughout its lifecycle.

## Conclusions

In this study, we have traced the dynamic transcriptional changes that occur throughout the lifecycle of the major helminth *F. gigantica* that allow the parasite to adjust to changing external environments and to both the intermediate invertebrate snail host and the definitive vertebrate mammalian host. In-depth analysis of the eight transcriptomes has elucidated key molecules and pathways important for each life-cycle stage, providing insight into *F. gigantica*-specific pathogenicity (Fig. 1). This data allowed the first opportunity for in-depth comparative analysis with *F. hepatica*, facilitating the investigation of transcription of stage-specific genes. This study and the availability of a unique and comprehensive transcriptomic data-set will lay the foundation for future studies to enhance our understanding of the similarities/differences between the *Fasciola* species, which may account for their host specificities.

Studies have shown that in areas where both *F. gigantica* and *F. hepatica* species are present such as China, India, Korea, Japan and Vietnam, these parasites have the ability to hybridize/introgress [52–57]. *F. gigantica* and *F. hepatica* parasites are hermaphrodites and have the ability to reproduce by self-fertilization as well as cross-fertilization. However, hybrid forms are more likely to be aspermic and multiply by parthenogenesis [58]. Current methods that differentiate between these two *Fasciola* species and the hybrid forms rely on analysis of mitochondrial markers (with limited nuclear markers) and do not provide an accurate reflection of the level of hybridization/introgression occurring within the hybrid forms [57, 59–61]. The availability of extensive *F. hepatica* genomic and transcriptomic sequencing coupled with proteomic studies [46] has improved our understanding of liver fluke biology and has driven the development of new panels of markers including SNPs [40] and microsatellites [62] for population genetic studies. Coupled with the present advances in our knowledge of *F. gigantica* transcriptomics described in this paper we can now progress investigations into the biology and pathogenicity of emerging *Fasciola* hybrids.

## Methods

### Parasite materials

Eggs of *F. gigantica* were collected from the gall-bladders of naturally infected buffaloes from the Guangxi Zhuang Autonomous Region (GZAR), PR China. The newly-hatched miracidia were obtained from eggs incubated at 29 °C for 11 days. To collect the snail-specific stages, *Galba pervia* snails were infected with *F. gigantica* miracidia (3–5 miracidia/snail). Rediae and cercariae were collected after 15 and 40 days post-infection (dpi), respectively. After 42 days of snail infection, the snails were shed to recover the metacercariae, the infective stage of the mammalian host. A total of 15 water buffaloes were infected orally with 500 viable metacercariae per animal in GZAR, PR China. At 42, 70 and 98 dpi, five infected buffaloes were slaughtered and the parasites recovered from the liver tissue and gall bladders (15 juvenile flukes at 42 dpi, 15 juvenile flukes at 70 dpi and 6 adult flukes at 98 dpi) [63]. The identification of *F. gigantica* was based on characteristic morphological features, which was further confirmed by amplification and sequencing of the internal transcribed spacer (ITS) region as previously described [60]. Each collected sample was washed > 10 times in physiological saline in order to remove any bacterial or host DNA contaminants and was snap frozen in liquid nitrogen followed by storage at − 80 °C for later use.

### RNA isolation, library preparation and transcriptome sequencing

Total RNA was extracted using TRIzol according to the manufacturer’s instructions (Invitrogen, USA) from three biological replicates of each *F. gigantica* lifecycle stage (pool of eggs comprising of *n* > 10,000; pool of miracidia comprising n > 10,000; pool of rediae comprising *n* > 200; pool of cercariae n > 200; pool of metacercariae comprising *n* > 5000; five juvenile flukes at 42 dpi; five juvenile flukes at 70 dpi and two adult *F. gigantica*). All RNA samples were treated with 20 units of RQ1 RNase-Free DNase (Promega, Madison, USA) to remove any residual genomic DNA according to the manufacturer’s instructions. RNA and sample library integrity and concentration were confirmed using the Bioanalyzer 2100 (Agilent Technologies) and the Qubit® RNA Assay Kit by a Qubit® 2.0 Fluorometer (Life Technologies, CA, USA). Library construction was performed according to the Illumina sample preparation for RNA-seq protocol. The poly (A) mRNA was isolated from total RNA using magnetic beads with Oligo (dT), followed by fragmentation and cDNA synthesis. The resulting short mRNA fragments were purified and resolved with ethidium bromide (EB) buffer for end reparation and single nucleotide A (adenine) and subsequent adapter addition. Sequencing of the library preparations was performed by Novogene Bioinformatics Technology Company Ltd., Beijing, China (www.novogene.cn) on an Illumina Hiseq X Ten platform to obtain paired-end reads.

### Assembly, annotation and gene expression analysis

Illumina HiSeq reads were trimmed to Q ≥ 30 and adaptors removed using in house Python scripts. De novo reconstruction of transcriptomes from RNA-Seq data was performed by constructing a k-mer dictionary (*k* = 25), and running the Trinity software according to the software instructions [64]. Briefly, the sequence data were partitioned into many individual de Bruijn graphs, each representing the transcriptional complexity at a given gene or locus. Each graph was processed independently to extract full-length splicing isoforms and to tease apart transcripts derived from paralogous genes. The final full-length transcripts were analysed by Corset to hierarchically cluster the contigs and compute the transcript length and distribution of sequences [65]. The sequences were aligned to the available *F. gigantica* genome sequences (Genbank MKHB00000000.1 [66];) to remove any sequences that originated from contaminating snail or buffalo tissue ingested by the parasites and/or extracted during parasite sample preparation (parameter settings: total score > 100, query cover > 95, e-value < 1 × e^− 10^).

The transcriptional expression of the unigenes within the lifecycle stage-specific transcriptomes was calculated using the RSEM program (v1.1.17 [67];). The alignments produced by Expectation-Maximization analysis resulted in digital expression levels for each unigene that were normalized to produce fragments per kb per million reads (FPKM [68];). The resulting *P* values were adjusted using the Benjamini and Hochberg’s approach for controlling the false discovery rate. Genes with an adjusted *P*-value < 0.05 as determined using DESeq were assigned as differentially expressed. Co-expression cluster analysis was carried out using Clust (v1.0.0 beta [69];), using default parameters of automatic normalization, cluster tightness of 1.0 and filtering of flat and low expression genes. Gene ontology enrichment analysis was carried out using hypergeometric tests using R. Further hierarchical clustering was carried out on those transcripts that displayed at least a 16-fold difference in expression between any of the lifecycle stages, represented by 9467 transcript clusters with a baseline cut-off of 1 FPKM in all three replicates, graphically represented using heatmaps generated using the R program, pheatmap.

Annotation of the *F. gigantica* transcripts was carried using the following in silico tools: (1) NCBI BLAST (v2.2.28; e-value ≤1 × e^− 5^) was used to interrogate the NCBI non-redunant (Nr) and nucleotide (Nt) databases, the KOG and COG databases and the protein sequence database, SwissProt; (2) Gene Ontology (GO) analysis using custom Perl Scripts (e-value ≤1 × e^− 6^) and Blast2GO v2.5 software [70]; (3) Analysis of protein conserved domain was carried using the Pfam database, using HMMER 3.0 (e-value ≤0.01). Any unigenes that did not align to any database were predicted by ESTScan software [71]. Stage-specific transcription was analysed using GO and KEGG databases, based on the stringent criteria of transcripts with < 1 FPKM in all three replicates to focus on the abundantly transcribed genes. Metabolic pathway analysis was carried out by normalizing the global patterns of expression at the KEGG module level by grouping the transcript clusters annotated by KEGG together per KEGG module/pathway, graphically represented using heatmaps generated using the R program, pheatmap.

### Comparative analyses with *Fasciola hepatica*

Reciprocal BLAST analyses were carried out using the *F. gigantica* DEG unigene dataset (58,422 transcript clusters) and the gene model dataset derived from the *F. hepatica* genome (22,676 gene models [40];), using a BLASTn (v2.2.29; e-value ≤1 × e^− 5^). Comparative gene ontology analysis was carried out on egg, metacercariae and adult transcriptomes of *F. gigantica* and *F. hepatica*. Enrichment of GO terms was determined using hypergeometric tests in R. Co-expression cluster analysis was carried out on the genes transcribed by the egg, metacercariae and adult stages of *F. gigantica* and *F. hepatica* using Clust (v1.0.0 beta [69];), using default parameters of automatic normalization, cluster tightness of 1.0 and filtering of flat and low expression genes. Gene ontology enrichment analysis was carried out using hypergeometric tests using R. The most abundantly expressed genes were determined from the transcriptional profile with the egg, metacercariae and adult parasites from *F. gigantica* (present study) and *F. hepatica* [40]. Reciprocal BLAST analyses were carried out using the most abundantly transcribed 250 transcripts from each transcriptome (BLASTn, v2.2.29; e-value ≤1 × e^− 5^). Circos diagrams were generated using Circos Table Viewer online [72], using the exponential value of the e-value comparison from BLASTn analysis.

### *F. gigantica* cathepsin cysteine proteases and asparaginyl endopeptidases (legumains)

Transcript clusters representing cathepsin L and B proteases and legumains were identified based on BLAST analyses (v2.2.29) using publically available sequences from NCBI (Additional file 11: Table S9), comparative analyses with the sequences annotated within the *F. hepatica* genome [40, 51] and parsing by putative annotation. The transcriptional profile of these sequences across the *F. gigantica* lifecycle was determined and analysed by hierarchical clustering graphically represented using heatmaps generated using the R program, pheatmap.

## Supplementary information

**Additional file 1: Table S1.** Enriched GO terms of the DEG from pairwise comparisons between the *Fasciola gigantica* lifecycle stages.

**Additional file 2: Table S2.** Unique GO terms associated with snail and buffalo-associated lifecycle stages.

**Additional file 3: Figure S1.** Cluster analysis of the unigenes transcribed across the *Fasciola gigantica* lifecycle based on gene expression. Sixteen clusters were generated by cluster analysis using Clust, shown by the individual graphs (C0-C15). The lifecycle stages are represented as follows: egg, mir: miracidia, red: rediae, cer: cercariae, met: metacercariae, 42d: juvenile fluke 42dpi. 70d: juvenile fluke 70dpi and ad: adult.

**Additional file 4: Table S3.** Gene ontology enrichment cluster analysis of the unigenes transcribed by eight *Fasciola gigantica* lifecycle stages.

**Additional file 5: Table S4.** List of the 34,264 *Fasciola gigantica* differentially expressed genes.

**Additional file 6: Table S5.** Enriched GO terms within egg, metacercariae and adult transcriptomes from comparative analysis between *Fasciola gigantica* and *Fasciola hepatica*.

**Additional file 7: Figure S2.** Comparative cluster analysis of gene transcription by *Fasciola gigantica* and *Fasciola hepatica* egg, metacercariae and adult lifecycle stages. Twelve clusters were generated by cluster analysis using Clust, shown by the individual graphs (C0-C11). The lifecycle stages are represented as follows: egg, met: metacercariae and ad: adult. The number of species-specific transcripts are shown, with the numbers in brackets representing the number of homologous sequences observed within each cluster.

**Additional file 8: Table S6.** Gene ontology enrichment cluster analysis of the co-expressed genes transcribed by *Fasciola gigantica* and *Fasciola hepatica* egg, metacercariae and adult lifecycle stages.

**Additional file 9: Table S7.** Number of the 9468 DEG displaying at least a 16-fold difference annotated with gene ontology terms relating to Molecular Function.

**Additional file 10: Table S8.** Comparative analysis of the top 250 most abundant *Fasciola gigantica* transcripts compared with homologous sequences in *Fasciola hepatica.*

**Additional file 11: Table S9.** GenBank accession numbers of the publically available *Fasciola gigantica* cathepsin L, cathepsin B and legumain genes.

## Data Availability

The *F. gigantica* transcriptome datasets supporting the conclusions of this article are available via NCBI BioProject: PRJNA350370. The *F. hepatica* datasets [40] used for the homology studies are freely available from European Nucleotide Archive under accessions LN627018-LN647175 (assembly data), PRJEB6687 (genomic read data) and PRJEB6904 (transcriptomic read data).

## Abbreviations

BLAST: Basic Local Alignment Search Tool
DEG: Differentially expressed genes
dpi: Days post infection
EB: Ethidium bromide
EVs: Extracellular vesicles
FABP: Fatty acid binding protein
FPKM: Fragment per kilobase per million reads
GO: Gene ontology
GST: Glutathione S-transferase
GZAR: Guangxi Zhuang Autonomous Region
HDM: Helminth defense molecule
ITS: Internal transcribed spacer
MAC: Membrane attack complex
NEJ: Newly excysted juveniles
PRX: Peroxiredoxin
QMF: Quantitative metabolic fingerprint
SNP: Single nucleotide polymorphisms
SOD: Superoxide dismutase
WHO: World Health Organisation
