# Supplementary figures and images for: Complex and dynamic transcriptional changes allow the helminth Fasciola gigantica to adjust to its intermediate snail and definitive mammalian hosts
Source: BMC Genomics. 2019 Oct 12;20:729. doi: 10.1186/s12864-019-6103-5 (PMC6790025; doi:10.1186/s12864-019-6103-5)

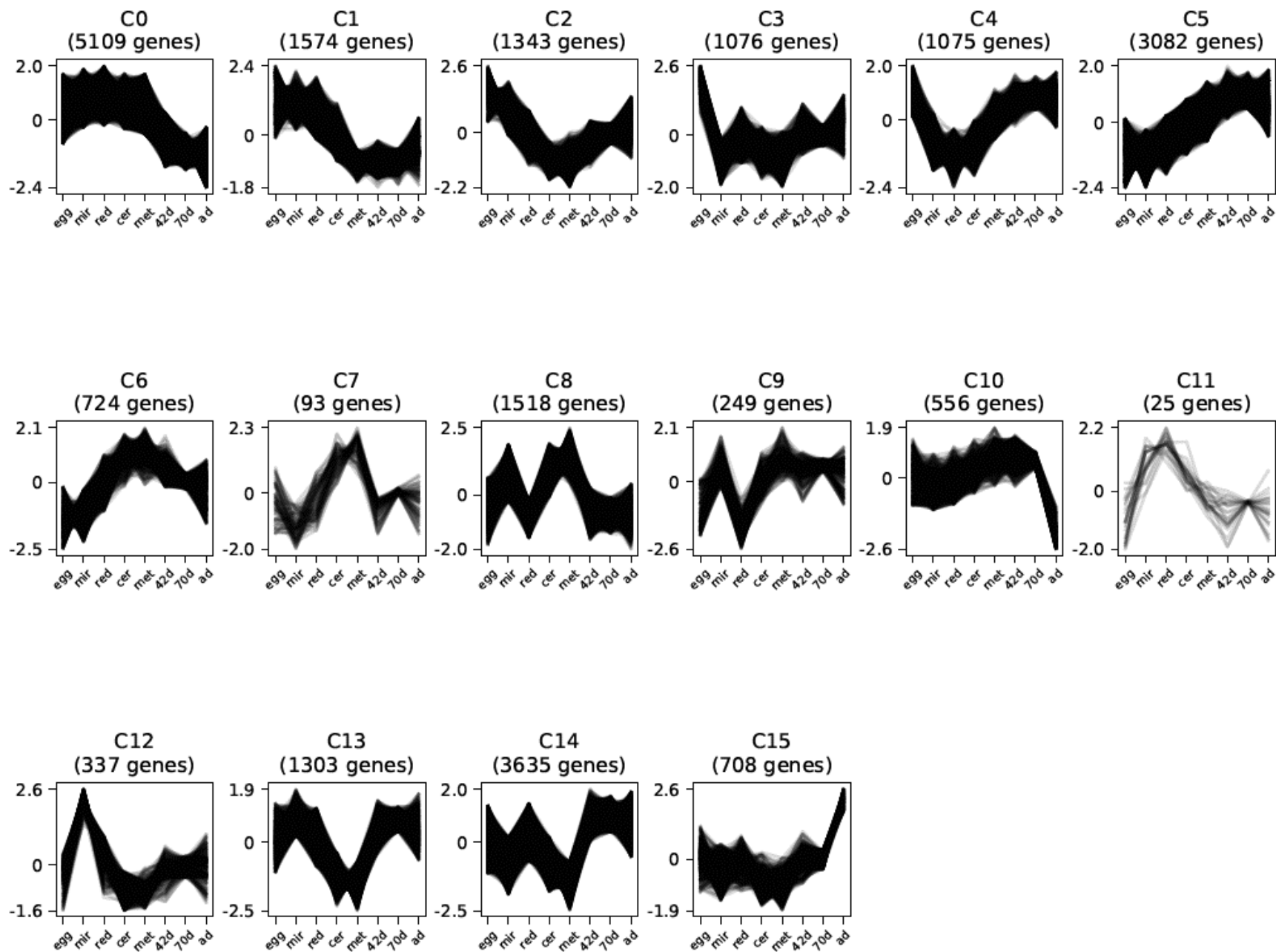

Supplement: Supplementary file 3 — Additional file 3: Figure S1. Cluster analysis of the unigenes transcribed across the Fasciola gigantica lifecycle based on gene expression. Sixteen clusters were generated by cluster analysis using Clust, shown by the individual graphs (C0-C15). The lifecycle stages are represented as follows: egg, mir: miracidia, red: rediae, cer: cercariae, met: metacercariae, 42d: juvenile fluke 42dpi. 70d: juvenile fluke 70dpi and ad: adult. [file 12864_2019_6103_MOESM3_ESM.pdf]

***F. hepatica******F. gigantica******F. hepatica******F. gigantica***

283 (62)

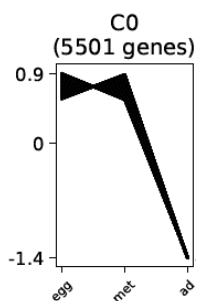

5218 (62)

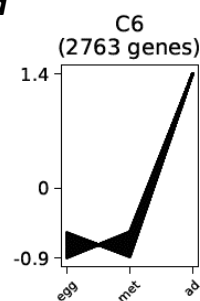

780 (104)

1983 (104)

500 (159)

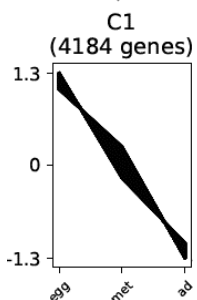

3684 (159)

1392 (197)

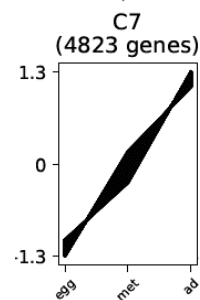

3431 (197)

344 (101)

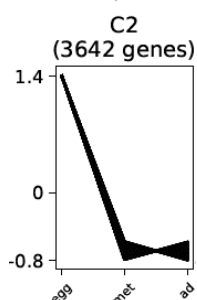

3298 (101)

1033 (228)

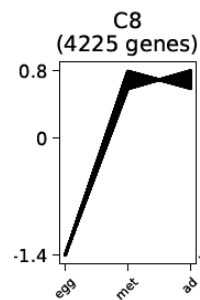

3192 (228)

545 (91)

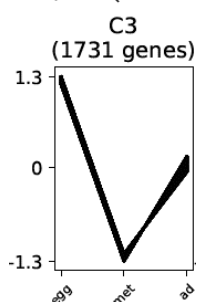

1186 (91)

723 (70)

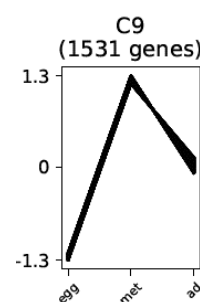

808 (70)

1047 (79)

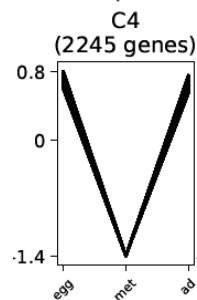

1198 (79)

443 (38)

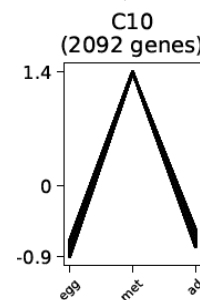

1649 (38)

1021 (82)

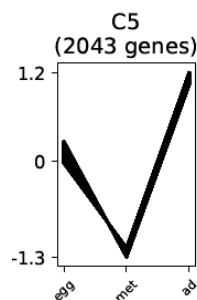

1022 (82)

400 (95)

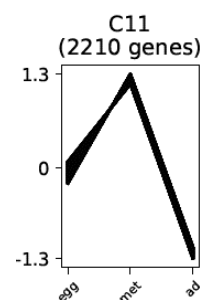

1810 (95)

Supplement: Supplementary file 7 — Additional file 7: Figure S2. Comparative cluster analysis of gene transcription by Fasciola gigantica and Fasciola hepatica egg, metacercariae and adult lifecycle stages. Twelve clusters were generated by cluster analysis using Clust, shown by the individual graphs (C0-C11). The lifecycle stages are represented as follows: egg, met: metacercariae and ad: adult. The number of species-specific transcripts are shown, with the numbers in brackets representing the number of homologous sequences observed within each cluster. [file 12864_2019_6103_MOESM7_ESM.pdf]
