# Supplementary material for: Complex and dynamic transcriptional changes allow the helminth Fasciola gigantica to adjust to its intermediate snail and definitive mammalian hosts
Source: BMC Genomics. 2019 Oct 12;20:729. doi: 10.1186/s12864-019-6103-5 (PMC6790025; doi:10.1186/s12864-019-6103-5)
Supplement: Supplementary file 11 — Additional file 11: Table S9. GenBank accession numbers of the publically available Fasciola gigantica cathepsin L, cathepsin B and legumain genes. [file 12864_2019_6103_MOESM11_ESM.docx]

**Additional file 11: Table S9: GenBank accession numbers of the publically available *Fasciola gigantica* cathepsin L, cathepsin B and legumain genes.**

| **Protease description** | **Accession Number** |
| --- | --- |
| Cathepsin L1 | AF112566 |
| Cathepsin L1 | AF239264 |
| Cathepsin L1 | AF239265 |
| Cathepsin L1 | AF239266 |
| Cathepsin L1 | AF419329 |
| Cathepsin L1 | AY428949 |
| Cathepsin L1 | EF536899 |
| Cathepsin L1 | JQ342985 |
| Cathepsin L2 | AF510856 |
| Cathepsin B1 | AY227673 |
| Cathepsin B2 | AY227674 |
| Cathepsin B3 | AY227675 |
| Cathepsin B4 | KM099341 |
| Cathepsin B5 | KT149795 |
| Cathepsin B7 | KT781072 |
| Legumain 1 | EF206821 |
| Legumain 2 | EF206822 |
